# Analysis of EEG features and study of automatic classification in first-episode and drug-naïve patients with major depressive disorder

**DOI:** 10.1186/s12888-023-05349-9

**Published:** 2023-11-13

**Authors:** Yuanyuan Huang, Yun Yi, Qiang Chen, Hehua Li, Shixuan Feng, Sumiao Zhou, Ziyun Zhang, Chenyu Liu, Junhao Li, Qiuling Lu, Lida Zhang, Wei Han, Fengchun Wu, Yuping Ning

**Affiliations:** 1 The First School of Clinical Medicine, Southern Medical University, Guangzhou, China; 2 Department of Psychiatry, The Affiliated Brain Hospital of Guangzhou Medical University, Guangzhou, China; 3 Department of Psychiatry, The Brain Hospital of Guangxi Zhuang Autonomous Region, Guangxi, China; 4 Guangdong Engineering Technology Research Center for Translational Medicine of Mental Disorders, Guangzhou, China; 5 Key Laboratory of Neurogenetics and Channelopathies of Guangdong Province and the Ministry of Education of China, Guangzhou Medical University, Guangzhou, China

**Keywords:** Major depressive disorder, Electroencephalogram, Machine learning, Automated classification

## Abstract

**Background:**

Major depressive disorder (MDD) has a high incidence and an unknown mechanism. There are no objective and sensitive indicators for clinical diagnosis.

**Objective:**

This study explored specific electrophysiological indicators and their role in the clinical diagnosis of MDD using machine learning.

**Methods:**

Forty first-episode and drug-naïve patients with MDD and forty healthy controls (HCs) were recruited. EEG data were collected from all subjects in the resting state with eyes closed for 10 min. The severity of MDD was assessed by the Hamilton Depression Rating Scale (HAMD-17). Machine learning analysis was used to identify the patients with MDD.

**Results:**

Compared to the HC group, the relative power of the low delta and theta bands was significantly higher in the right occipital region, and the relative power of the alpha band in the entire posterior occipital region was significantly lower in the MDD group. In the MDD group, the alpha band scalp functional connectivity was overall lower, while the scalp functional connectivity in the gamma band was significantly higher than that in the HC group. In the feature set of the relative power of the ROI in each band, the highest accuracy of 88.2% was achieved using the KNN classifier while using PCA feature selection. In the explanatory model using SHAP values, the top-ranking influence feature is the relative power of the alpha band in the left parietal region.

**Conclusions:**

Our findings reveal that the abnormal EEG neural oscillations may reflect an imbalance of excitation, inhibition and hyperactivity in the cerebral cortex in first-episode and drug-naïve patients with MDD. The relative power of the alpha band in the left parietal region is expected to be an objective electrophysiological indicator of MDD.

**Supplementary Information:**

The online version contains supplementary material available at 10.1186/s12888-023-05349-9.

## Background

Major depressive disorder (MDD) is a debilitating disease that is characterized by noticeable alterations in mood, interest, pleasure, cognition, psychomotor activity and vegetative symptoms [1]. The disease can have a negative impact on people’s daily lives, and the number of people suffering from the disease is increasing yearly. The current diagnosis of MDD is dependent on clinical interviewing with patients, which has obvious disadvantages, including poor sensitivity, patient denial, subjective biases and inaccuracy. Detecting the declines in brain physiology prior to the onset of subjective symptoms plays a significant role in the recognition of MDD at an early stage, thus allowing early treatment that is beneficial for MDD patients.

Electroencephalography (EEG) has been characterized as having high temporal resolution and low cost, which makes it more feasible for clinical application [2]. It contributes to the categorization of the electrophysiology of the brain in symptomatic and asymptomatic individuals. Pizzagalli et al. showed that depressive subjects exhibited more β3 in the pronounced right inferior and superior frontal regions than healthy control subjects and less β3 in the posteromedial cluster, including the posterior cingulate cortex and precuneus cortex [3]. Arikan et al. found that the group with suicidal ideation showed significantly higher high-gamma power (40–50 Hz) than other groups through rest-state EEG [4]. A recent study recruiting 44 late-life depression (LLD) patients and 41 healthy controls (HCs) showed that LLD patients had higher beta frequency activity and increased alpha activity than the HC group, and there were no correlations between beta power and the severity of MDD [5]. Because of the heterogeneity of patients and the influence of drug medication, the characteristics of EEG spectrum power in first-episode MDD remain unclear.

From a neuropsychiatric viewpoint, there is a growing awareness that MDD is characterized by disrupted connectivity between cortical and subcortical brain regions. There is evidence that synchronous oscillations can modulate functional connectivity between different brain regions. Liu et al. demonstrated that the dysfunction of oscillatory networks may be a promising indication of the pathoconnectomics of MDD [6]. A study of 16 depressive patients and 14 healthy controls found that depressive patients had abnormally enhanced brain functional connectivity in the gamma band by computing EEG coherence and demonstrated that the gamma band was sensitive to emotion processing [7]. Li et al. demonstrated that alpha, theta and delta phase synchronization were decreased, but beta phase synchronization was increased in depressive patients, which indicated that underphased synchronization and asynchrony during working memory processing reveal the impairment of attention efficiency, memory and cortical inhibition in patients with MDD [8]. Previous studies focused on cognitive tasks and rest-state EEG can provide more informative insights into the pathophysiology of MDD. Therefore, it is essential to investigate the changes in scalp functional connectivity in the resting state in MDD patients without any drug medication, and the correlation with psychiatric symptoms needs to be further explored.

Recently, EEG-based machine learning (ML) techniques have attracted a considerable amount of attention due to their ability to noninvasively explore neuroimaging data to build computer-aided diagnosis solutions to advance the diagnosis of MDD. Bachmann et al. recruited 13 drug-free outpatients with MDD using linear methods and nonlinear methods to discriminate depressive patients and healthy controls. The results showed that combinations of these features had a maximal classification accuracy of 92% [9]. One study utilized 6 channels (FT7, FT8, T7, T8, TP7, TP8) with the Gaussian kernel of SVM and achieved an accuracy of 96.02% for screening depression [10]. However, no study has used machine learning to combine spectral power and scalp functional connectivity to identify MDD in the early stage.

To date, most studies focusing on the brain spectral signature of MDD have shown inconsistent results, and no study has examined first-episode and drug-naïve MDD with machine learning approaches combining spectral power and scalp functional connectivity. To address the heterogeneity of the results of electroencephalography in MDD studies, we collected detailed data on whole-brain neural activity and functional connectivity to (1) determine the specific electrophysiological indicators in first-episode and drug-naïve patients with MDD and (2) explore the role of these electrophysiological indicators in the clinical diagnosis of MDD using machine learning.

## Methods and materials

### Participants

Forty right-handed patients with first-episode and drug-naïve MDD and forty right-handed HCs were recruited to participate in this study. All MDD patients were outpatients or inpatients at the Affiliated Brain Hospital of Guangzhou Medical University from June 2021 to November 2022. All HCs participants were recruited from the community through posters or forums.

The inclusion criteria for patients were as follows: (1) met the Diagnostic and Statistical Manual of Mental Disorders (DSM-V) criteria for MDD; (2) age 18–45 years; (3) the duration of the illness was less than 2 years; (4) no history of psychotic medication or any other somatic therapy (e.g., modified electric convulsive treatment); and 4) the value of the HAMD-17 scale ≥ 17. The inclusion criteria for HCs were as follows: (1) no psychiatric disorders meeting the DSM-V criteria; and (2) no family history of mental disease. The exclusion criteria for all participants were as follows: (1) had serious physical and neurological disease; (2) had a history of epilepsy, febrile convulsions or coma; (3) had history of substances and drug abuse; and (4) could not cooperate with EEG examination.

The study was approved by the Ethics Committee of the Affiliated Brain Hospital of Guangzhou Medical University. The study was conducted according to the Declaration of Helsinki. All participants signed an informed consent form ahead of enrollment.

### Clinical measurement

All participants were interviewed and underwent a structured clinical interview by two experienced psychiatrists using the Mini-International Neuropsychiatric Interview (M.I.N.I.). The severity of MDD was assessed using the Hamilton Depression Rating Scale (HAMD-17).

### EEG acquisition and processing

#### EEG Recording

Resting-state EEG data were recorded for each participant with their eyes closed for ten minutes by a trained technician. EEG data were obtained from 32 channels of Ag/AgCl electrodes using the Neuroscan system with a sampling rate of 1000 Hz and paced on the scalp in accordance with the International 10–20 system: C3, C4, F3, F4, F7, F8, Fp1, Fp2, FC1, FC2, FC5, FC6, Fz,T7, T8, P3, P4, Pz, CP1, CP2, CP5, CP6, Oz, P7, P8, O1, O2, PO3, PO4. During EEG recording, the impedance of each electrode was maintained below 10 KΩ, and Pz was set as the reference electrode.

#### EEG Preprocessing

All EEG data were imported into the EEGLAB toolbox in MATLAB R2013a software. The bandpass filter band was 0.1–80 Hz, and the notch filter band range was 49–51 Hz. The continuous EEG was downsampled to 500 Hz. A 2-s epoch segment was set for bad epoch data rejection and further analysis [11]. Bad channels were interpolated using spherical splines. Independent component analysis was used to remove eye movement artifacts. Segments with voltage values exceeding ± 80 were manually rejected. EEG data were rereferenced to the common average reference. Spectral power and functional connectivity in the 2-s segments were measured. Finally, we excluded four EEGs from MDD patients and nine EEGs from HCs for their poor quality.

### Power Spectrum

The power spectrum belonged to quantitative EEG, which simply reflected the changes of activity of the brain. Moreover, the power spectrum was regarded as linear parameters which were used in many existed studies. Power spectrum was calculated by performing a Fast Fourier Transform (FFT) algorithm to extract the features in the frequency domain, including: alpha (8–13 Hz), beta (13–30 Hz), theta (4–8 Hz), gamma (30–80 Hz), delta (1–4 Hz). The scalp electrodes were clustered into eight groups according to the regions of interest (ROIs), including the right parietal region (C4, CP6, CP2, P4), right frontal region (FC2, FC6, F4, FP2), right occipital region (PO4, O2), right temporal region (F8, T8, P8), left parietal region (P3, CP1, CP5, C3), left frontal region (FP1, F3, FC5, FC1), left temporal region (P7, T7, FT7), left occipital region (O1, PO3), and left parietal region (C3, CP5, CP1, P3). The relative power in each frequency band was derived by dividing the absolute power of each frequency band by the total broadband of the absolute power, which included the delta band, theta band, alpha band, beta band and gamma band. The formulas were as follows:

The relative of alpha power = the absolute power of alpha / (the absolute power of delta + the absolute power of theta + the absolute power of alpha + the absolute power of beta + absolute power of gamma).

### Functional connectivity

The weighted phase lag index (wPLI) was used to compute the functional connectivity between all pairwise combinations of 29 channels. The connectivity between the two regions was calculated by the average wPLI value of all electrode pairs of recording sites between the two regions.

### Machine learning analysis

Python was used to conduct the data analysis of the five classification algorithms, including decision tree (DT), gradient boosting decision tree (GBDT), support vector machine (SVM), naïve Bayesian (NB) and K-nearest neighbor (KNN). We used three methods to select features, including recursive feature elimination based on SVM (SVM-RFE), selection operator and least absolute shrinkage based on L1 (LASSO-L1) and principal component analysis (PCA).

The relative power of each band of each scalp area was regarded as set (1) The functional connectivity of any two scalp areas was regarded as set (2). The combinations of set 1 and set 2 were regarded as set (3). Permutation testing was utilized to evaluate the statistical significance of the classifier performance.

The importance of each feature was interpreted by the SHAP values. SHAP is an acronym for Shapley Additive exPlanations, which was a unified framework introduced for interpreting machine learning predictions. The SHAP technique calculates the Sharpley value by evaluating the contribution and influence of each feature to model prediction [12]. The importance ranking of the top 20 factors with stability and interpretation using optimal model. The red part in feature value represents higher value. The higher SHAP value of a feature is given, the more important feature in predicting depression.

**Fig. 1:**
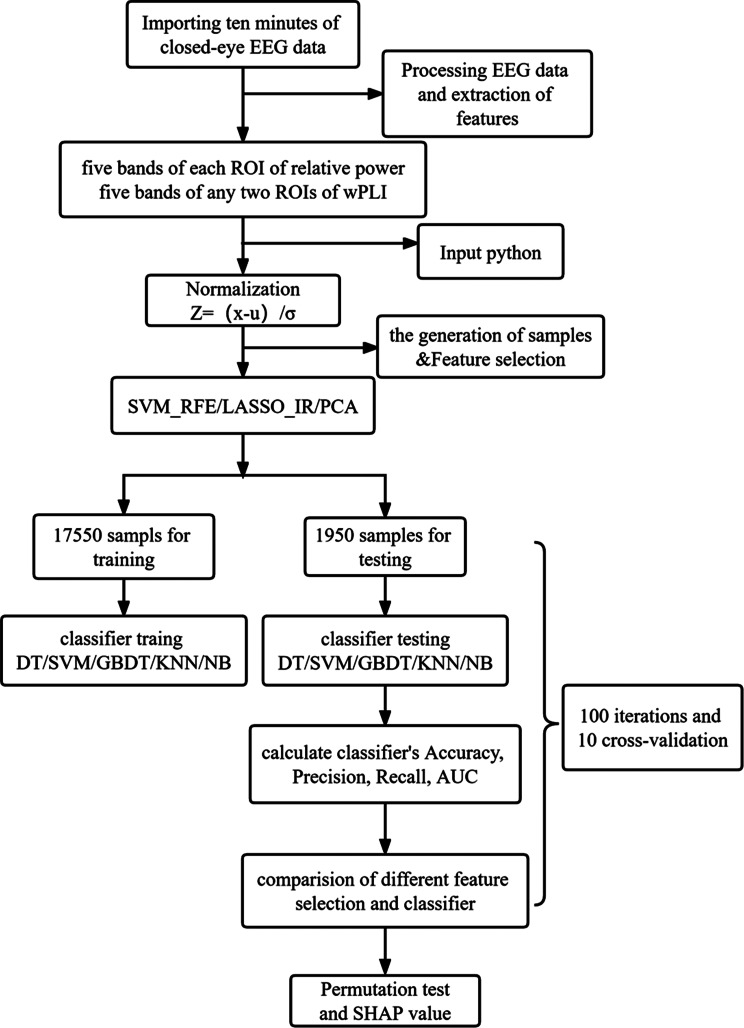
The flowchart of machine learning

As shown in Fig. 1, we used 1000 sampling points as a sample. Since the data sampling rate was down sampled to 500 Hz, a sample should take 2 s. After the data cutting, the total sample size of the two groups was 19,500. Then, 90% of the data was used for training and 10% for testing. The stratification of the dataset was based on the participants.

### Statistical analysis

Statistical analyses were carried out using SPSS (version 23.0). For continuous variables, an independent samples t-test was conducted to compare intergroup differences. The comparison of two groups of relative power was computed using the independent sample two-tailed t-test across ROIs in five frequency bands. Pearson’s χ^2^ test was used to test sex differences between two groups. The false-discovery rate (FDR) correction was employed to reduce the type I error in multiple comparisons. Pearson correlation analysis was performed to explore the correlations between EEG features and clinical factors. Results were defined as significant if the two-tailed p value was lower than 0.05.

## Results

### Demographic characteristics

Table 1 includes demographic information of the subjects in the MDD group and HC group. There was no significant difference between the two groups in terms of sex and age.

**Table 1:** Demographic characteristics of two groups

	MDD group (n = 36)	HC group (n = 31)	P-value
Gender(male/female)	14/22	14/17	0.590
Age(years)	22.7 ± 2.1	25.2 ± 6.9	0.054
Education (years)	15.0 ± 1.7	14.6 ± 2.5	0.500
Age of onset (years)	24.5 ± 7.6	—	—
Course of disease (month)	6(1,48)	—	—
HAMD-17	21.9 ± 6.9	—	—

MDD: major depression disorder; HC: Healthy control

### EEG feature analysis

#### The relative power

**Fig. 2:**
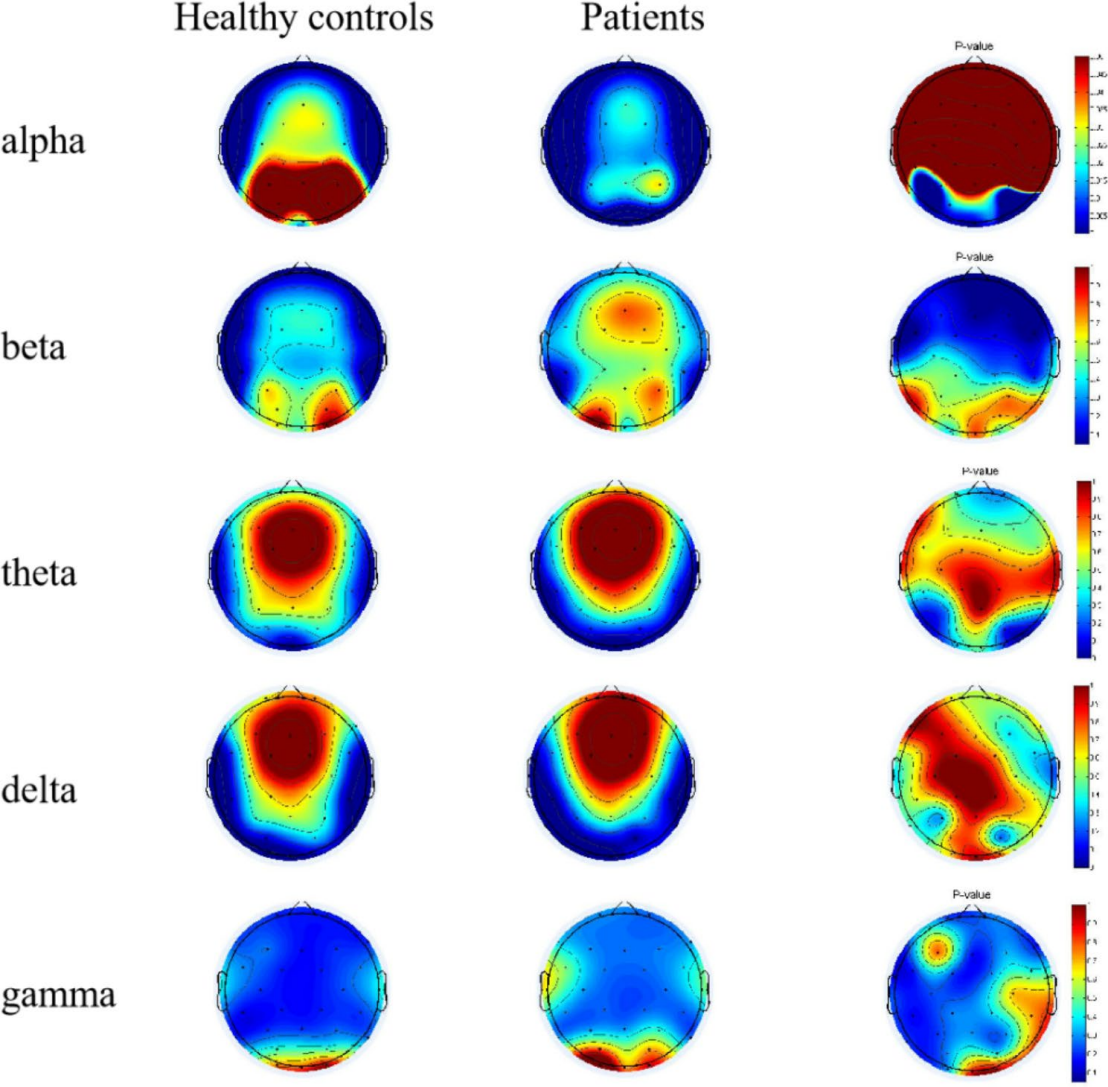
Comparison of the relative power between two groups in each ROI area in each frequency band

**Fig. 3:**
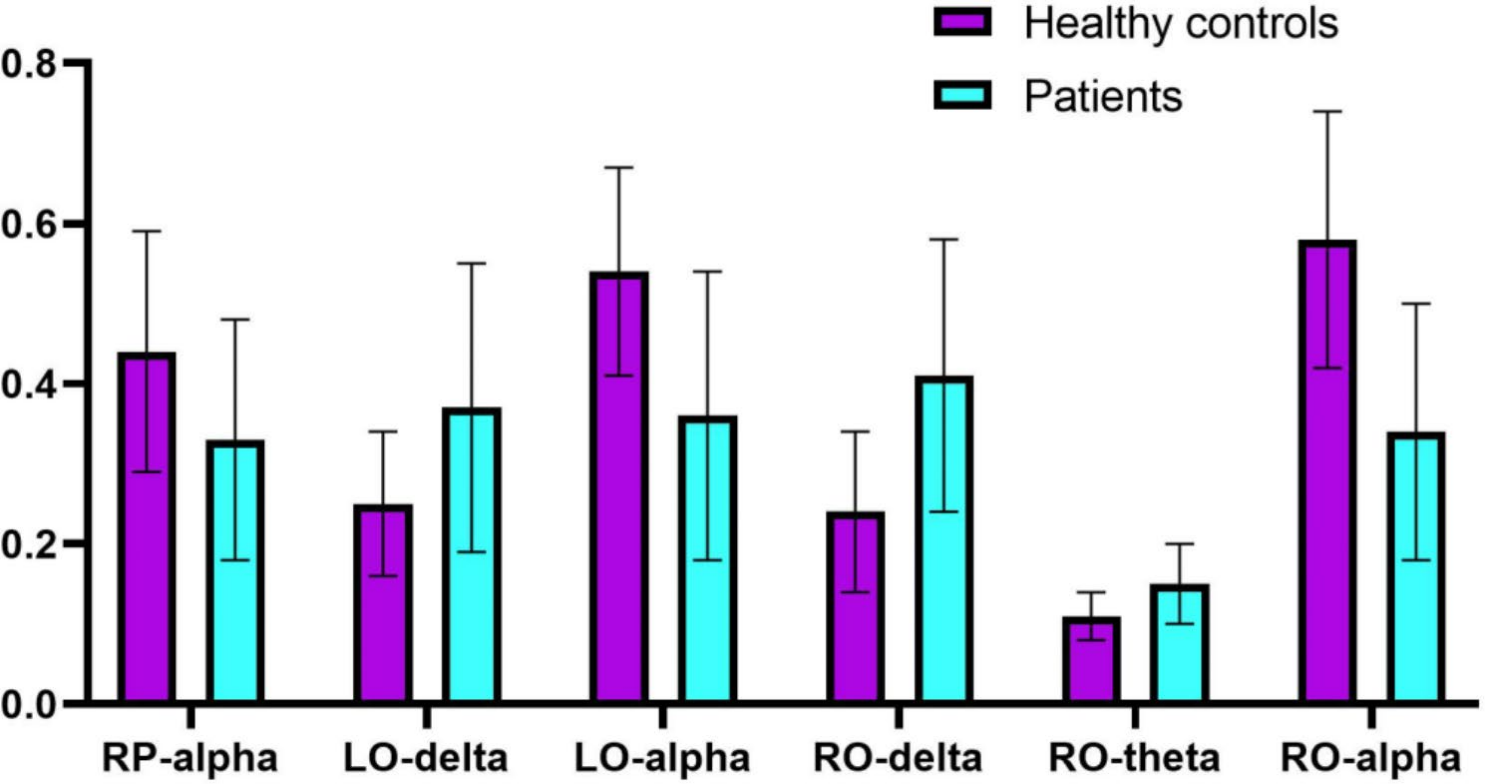
The differences in the relative power in frequency bands between the two groups (RP-alpha: Right Parietal-alpha; LO-delta: Left Occipital-delta; LO-alpha: Left Occipital-alpha; RO-delta: Right Occipital-delta; RO-theta: Right Occipital-theta; RO-alpha: Right Occipital-alpha)

As shown in Fig. 2, in the right posterior occipital region, the relative power of the delta and theta bands was found to be significantly different. The relative power of the alpha band was also significantly different in the whole posterior occipital region. There were no differences in beta and gamma bands. Furthermore, the relevant data were extracted. In the left occipital region, the relative power of the alpha band in the MDD group was significantly lower than that in the HC group *(t = 4.829, FDRp = 0.01)*, but the relative power of the delta band was significantly higher than that in the HC group *(t = -3.357, FDRp = 0.01).* In the right occipital region, the relative power of delta *(t = -4.934, FDRp = 0.01)* and theta *(t = -4.007, FDRp = 0.01)* bands in the MDD group was significantly higher than that in the HC group. The relative power of the alpha band in the MDD group was significantly lower than that in the HC group *(t = 6.142, FDRp = 0.01)*. In the right parietal region, the relative power of the alpha band in the MDD group was significantly lower than that in the HC group *(t = 2.886, FDRp = 0.03)* (Fig. 3).

### Functional connectivity

**Fig. 4:**
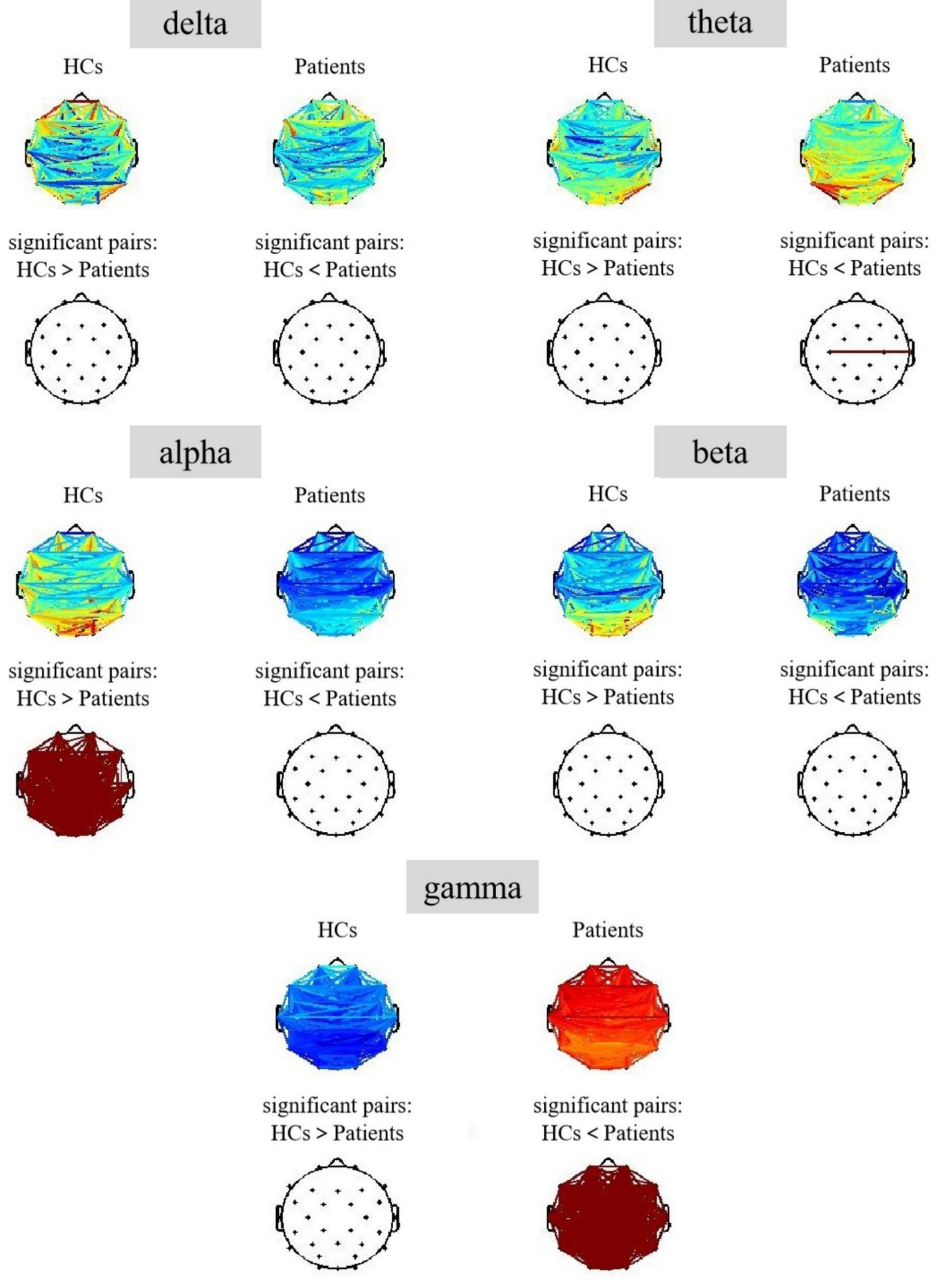
The functional connectivity of scalp brain regions between the two groups in each frequency band (significant pairs: Functional connectivity pairs representing two groups whose functional connectivity values are still statistically different after FDR correction; HCs > Patients: The functional connectivity was greater in HCs than in patients; HCs < Patients: The functional connectivity was weaker in HCs than in patients)

As shown in Fig. 4, there were no differences in wPLIs in the delta and theta bands. However, wPLIs were significantly weakened in the alpha band and enhanced in the gamma band across all regions of the brain in the MDD group (all p < 0.001).

### Correlations between EEG features and clinical symptoms

There were no correlations between the relative power of alpha and delta and HAMD-17 scores in the left occipital region (r = 0.201, p = 0.379; r = -0.233, p = 0.171) and right occipital region (r = -0.182, p = 0.289; r = -0.110, p = 0.521).

### Classification of MDD based on EEG features

The classification results of each feature set based on different classifiers with different feature selection methods were shown as Accuracy, Precision, Recall and AUC. They were expressed as mean ± sd (Table 2).

**Table 2:** The classification results of different sets

Feature selection	Model	Accuracy	Precision	Recall	AUC	*p*-value
**Set1**						
SVM-RFE	DT	0.751 ± 0.017	0.774 ± 0.016	0.773 ± 0.013	0.749 ± 0.017	0.0099
	SVM	0.776 ± 0.010	0.787 ± 0.013	0.809 ± 0.012	0.774 ± 0.010	0.0099
	GBDT	0.803 ± 0.013	0.817 ± 0.014	0.817 ± 0.013	0.800 ± 0.012	0.0099
	NB	0.679 ± 0.014	0.741 ± 0.019	0.627 ± 0.013	0.681 ± 0.014	0.0099
	KNN	0.772 ± 0.014	0.731 ± 0.014	0.913 ± 0.012	0.755 ± 0.015	0.0099
LASSO-LR	DT	0.830 ± 0.012	0.843 ± 0.013	0.846 ± 0.013	0.828 ± 0.012	0.0099
	SVM	0.859 ± 0.012	0.896 ± 0.011	0.837 ± 0.022	0.861 ± 0.010	0.0099
	GBDT	0.826 ± 0.008	0.845 ± 0.013	0.831 ± 0.013	0.827 ± 0.008	0.0099
	NB	0.661 ± 0.013	0.778 ± 0.018	0.525 ± 0.016	0.675 ± 0.012	0.0099
	KNN	0.851 ± 0.012	0.811 ± 0.013	0.946 ± 0.010	0.842 ± 0.013	0.0099
PCA	DT	0.792 ± 0.014	0.808 ± 0.015	0.809 ± 0.020	0.791 ± 0.015	0.0099
	SVM	0.870 ± 0.009	0.891 ± 0.011	0.867 ± 0.015	0.871 ± 0.010	0.0099
	GBDT	0.813 ± 0.013	0.832 ± 0.017	0.823 ± 0.015	0.813 ± 0.013	0.0099
	NB	0.687 ± 0.013	0.768 ± 0.013	0.608 ± 0.027	0.694 ± 0.013	0.0099
	KNN	0.882 ± 0.006	0.847 ± 0.008	0.955 ± 0.011	0.877 ± 0.008	0.0099
**Set2**						
SVM-RFE	DT	0.543 ± 0.034	0.596 ± 0.034	0.577 ± 0.047	0.541 ± 0.035	0.0099
	SVM	0.590 ± 0.037	0.597 ± 0.022	0.845 ± 0.042	0.560 ± 0.037	0.0099
	GBDT	0.598 ± 0.027	0.615 ± 0.019	0.764 ± 0.035	0.580 ± 0.025	0.0099
	NB	0.513 ± 0.029	0.609 ± 0.041	0.353 ± 0.064	0.532 ± 0.026	0.0099
	KNN	0.561 ± 0.017	0.576 ± 0.012	0.817 ± 0.033	0.527 ± 0.019	0.0099
LASSO-LR	DT	0.553 ± 0.024	0.599 ± 0.026	0.602 ± 0.033	0.544 ± 0.027	0.0099
	SVM	0.618 ± 0.032	0.622 ± 0.021	0.822 ± 0.028	0.594 ± 0.033	0.0099
	GBDT	0.585 ± 0.020	0.606 ± 0.013	0.748 ± 0.044	0.579 ± 0.046	0.0099
	NB	0.503 ± 0.026	0.607 ± 0.028	0.296 ± 0.073	0.524 ± 0.021	0.0099
	KNN	0.564 ± 0.019	0.573 ± 0.013	0.854 ± 0.036	0.521 ± 0.022	0.0099
PCA	DT	0.536 ± 0.030	0.585 ± 0.028	0.576 ± 0.049	0.524 ± 0.032	0.0099
	SVM	0.617 ± 0.039	0.615 ± 0.028	0.837 ± 0.025	0.579 ± 0.042	0.0099
	GBDT	0.575 ± 0.032	0.601 ± 0.019	0.730 ± 0.035	0.560 ± 0.027	0.0099
	NB	0.582 ± 0.020	0.623 ± 0.018	0.650 ± 0.048	0.575 ± 0.021	0.0099
	KNN	0.553 ± 0.025	0.571 ± 0.018	0.825 ± 0.039	0.517 ± 0.028	0.0099
**Set3**						
SVM-RFE	DT	0.721 ± 0.015	0.787 ± 0.019	0.723 ± 0.020	0.719 ± 0.011	0.0099
	SVM	0.755 ± 0.012	0.753 ± 0.010	0.768 ± 0.005	0.719 ± 0.012	0.0099
	GBDT	0.786 ± 0.011	0.768 ± 0.12	0.808 ± 0.011	0.778 ± 0.014	0.0099
	NB	0.639 ± 0.013	0.732 ± 0.021	0.590 ± 0.012	0.655 ± 0.016	0.0099
	KNN	0.758 ± 0.012	0.708 ± 0.019	0.865 ± 0.020	0.734 ± 0.017	0.0099
LASSO_LR	DT	0.828 ± 0.011	0.835 ± 0.018	0.833 ± 0.012	0.802 ± 0.012	0.0099
	SVM	0.837 ± 0.013	0.876 ± 0.008	0.822 ± 0.003	0.842 ± 0.015	0.0099
	GBDT	0.811 ± 0.012	0.832 ± 0.011	0.822 ± 0.022	0.838 ± 0.022	0.0099
	NB	0.661 ± 0.009	0.789 ± 0.012	0.588 ± 0.011	0.655 ± 0.008	0.0099
	KNN	0.844 ± 0.013	0.798 ± 0.018	0.900 ± 0.011	0.802 ± 0.021	0.0099
PCA	DT	0.786 ± 0.016	0.819 ± 0.012	0.758 ± 0.019	0.788 ± 0.016	0.0099
	SVM	0.845 ± 0.011	0.887 ± 0.014	0.856 ± 0.011	0.886 ± 0.012	0.0099
	GBDT	0.822 ± 0.012	0.844 ± 0.017	0.818 ± 0.011	0.823 ± 0.019	0.0099
	NB	0.667 ± 0.011	0.768 ± 0.023	0.668 ± 0.015	0.688 ± 0.011	0.0099
	KNN	0.876 ± 0.018	0.833 ± 0.012	0.923 ± 0.015	0.857 ± 0.012	0.0099

AUC: Area Under the Cure; *p*-value: Permutation test p value; SVM-RFE: Support Vector Machines- Recursive Feature Elimination; LASSO_LR: Least Absolute Shrinkage and Selection Operator- Logistic Regression; PCA: Principal Component Analysis; DT: Decision Tree; GBDT: Gradient Boosting Decision Tree; NB: Naïve Bayesian; KNN: K-Nearest Neighbor

The highest classification accuracy was achieved when only feature set 1 was used. That is, when we selected the relative power of each band of the ROI, we achieved the highest accuracy (88.2%) using PCA feature selection and the KNN classifier. The result of permutation test was less than 0.01. This result indicated that the prediction accuracy of the KNN classifier was significantly higher than that of the random prediction case, which is of practical significance.

**Fig. 5:**
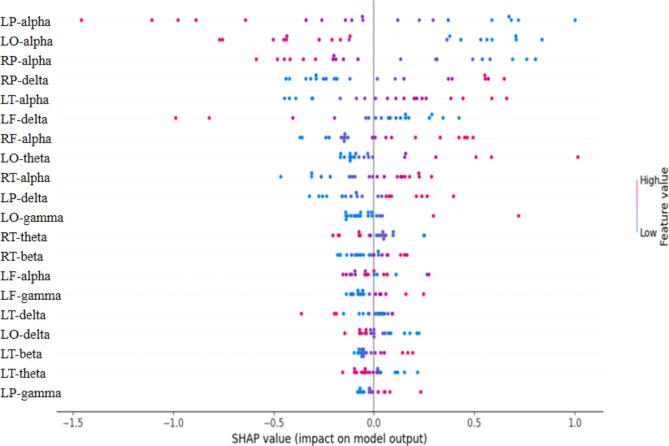
SHAP model explanation

Figure 5 shows the SHAP values of each feature for each sample. One dot represents a sample, and the color indicates the feature value (the blue color indicates a low value, and the red color indicates a high value). In the model, the top-ranking influence feature is the relative power of the alpha band in the left parietal (LP-alpha).

(LF/RF/LP/RP/LT/RT/LO/RO-delta/theta/alpha/beta/gamma: Left Frontal/ Right Frontal/ Left Parietal/ Right Parietal/ Left Temporal/ Right Temporal/ Left Occipital/ Right Occipital-delta/ theta/ alpha/ beta/ gamma).

## Discussion

This was the first study to use machine learning combined with spectral power and scalp functional connectivity to identify first-episode and drug-naïve MDD. The findings of our study included the following: (1) compared to the HC group, the relative power of the low delta and theta bands was significantly higher in the right occipital region, and the relative power of the alpha band in the entire posterior occipital region was significantly lower in the MDD group; (2) in the MDD group, the alpha band scalp functional connectivity was overall lower, while the scalp functional connectivity in the gamma band was significantly higher than that in the HC group; and (3) the relative power of each frequency band in each scalp brain region used as the input feature set achieved the highest accuracy rate (88.2%), and the top-ranking influence feature is the relative power of the alpha band in the left parietal region.

Our results showed that the relative power of the theta and delta bands in the MDD group was significantly higher than that in the HC group. Previous studies have also confirmed that the severity of MDD symptoms is positively proportional to theta power. The activity of the theta band is associated with emotional stimuli and that the activity of theta rhythms can be enhanced by high arousal from negative pictures [13], which may explain that the theta rhythm is associated with the electrical activity of the medial prefrontal cortex [14]. Lee et al. found that compared to the group with lower suicidal ideation scores, the relative power of theta in the central frontal regions (CZ, FZ, FCz, and F3) was significantly higher in the group with high suicidal ideation scores [15]. Delta waves affected the incentive and reward areas of the cerebral cortex, which suggested that they were significantly associated with emotion production [16]. It has been suggested that the high activity of the delta band in MDD patients is a compensatory mechanism for the deficits in cortical function caused by MDD, which is caused by MDD may be achieved through the modulation of cognitive function. An increase in the power of the delta band may inhibit interference affecting cognitive tasks in the cerebral cortex, which increases the attention and concentration required to perform certain cognitive tasks in patients with MDD [17]. However, our findings focused on the right occipital area, while there is no basis to support that the abnormal activation in this brain region is associated with the relative power enhancement of the delta and theta bands of the brain region. Further research is needed to investigate this topic.

Our findings also suggested that patients with MDD have lower relative alpha power in the occipital region than the HC group, which was consistent with previous studies [18–20]. Alpha rhythms are thought to be generated by cortico-thalamic brain interactions, which involve a large number of cognitive operations, particularly sensory system control, working memory and attention [21]. The decline in the relative power of the EEG alpha band in MDD reflected increasing negative emotions and significant activation of the cerebral cortex [22, 23], which may be related to a reduced thalamocortical synchronization system. Additionally, decreased alpha energy in the occipital region of depressed patients may be related to increased arousal and cortical excitability in the posterior occipital region of the brain, which revealed a chronic stress response in the development of MDD. Moreover, it may also be related to the genotype of the individual. Compared to gene Val carriers, MDD gene Met carriers had lower overall absolute alpha power under closed-eye conditions [20]. This may be because brain-derived neurotrophic factor (BDNF) protein secretion was limited in Met allele carriers [24] and the effect on functional connectivity. However, some studies have shown the opposite results [25, 26]. One reason may be related to the age of onset of the subjects. Alpha oscillations are prone to change during critical neural development, such as puberty [27]. Another explanation can be found in the heterogeneity of subjects, for example, the differences in drugs, sex, and subtypes of MDD. These factors should be more clearly controlled in the future. Significant changes in alpha rhythm can help explain the neural mechanisms of MDD, and our study – which is the first to examined untreated subjects – provides theoretical support for subsequent studies on neural marker identification and detection methods.

MDD is characterized by imbalances in communication between large-scale functional networks, including resting-specific hyper and hypoconnectivity within and between brain networks [28]. Our study demonstrated functional connectivity disorder between cortical regions in patients with MDD, which was consistent with previous studies [29, 30]. Previous studies have shown that MDD exhibits significantly disrupted network characteristics, which are closely related to the emotional processing disorder of MDD. That is, changes in the MDD network in the alpha band may lead to changes in emotional response or emotional arousal [30]. Alpha rhythm synchronization was associated with the activity of the default mode network (DMN) [31]. Thus, any disruption of the DMN in MDD may manifest as changes in EEG alpha oscillations and desynchronization of alpha band connections. However, some studies with other indicators of functional connectivity(e.g., coherence) have produced inconsistent results.A study of 12 outpatients with drug-free MDD and 10 HCs showed that MDD had significantly increased functional connectivity in the alpha and theta bands by using the signal synchronization called the “structural synchronization index“ [32]. Leuchter et al. found that patients with MDD had higher connectivity in beta, alpha, theta and delta bands than HCs [33]. Therefore, there is a need to use a uniform functional connectivity evaluation index to explore phase-synchronous changes in the alpha band of MDD with a large sample.

Our study showed that there existed a significant increase in functional connectivity between overall scalp-brain regions in the gamma band, which was in line with previous studies [7, 32, 34]. Previous studies confirmed that gamma oscillations are sensitive to emotional processing [7]. When patients were depressed, their brains became information activated or overloaded. It may be attributed to the fact that increased gamma functional connectivity leads to abnormal activation of the depressed brain or overload of communication. The increasing connectivity in the gamma band may reflect increased attentional function [35, 36], which reflect the neural mechanisms of information connectivity. Moreover, the functional connectivity enhancement of the gamma band in patients with MDD may be related to the relationship between serotonin and γ-aminobutyric acid (GABA) signaling. Serotonin is a widely distributed neurotransmitter in the central nervous system [37]. Previous studies have demonstrated that activation of 5-hydroxytryptamine signaling has been shown to inhibit the inhibitory function of GABA-energic neurons [38]. In 5-hydroxytryptamine deficiency (e.g., MDD), GABA-energic signaling in the cortex may be enhanced, and GABA plays a role in the production and regulation of endogenous motor cortical rhythm beta and gamma activity [39]. However, there are other results that may differ from our study. An EEG study showed that depressed patients in the beta band of the DMN showed greater functional connectivity [40]. Kim et al. suggested that the pathophysiological mechanisms of depressive states may be related to excessive neural processing in the beta band [41]. Therefore, further longitudinal studies with large samples are needed to investigate gamma band alterations in MDD.

It is worth mentioning that the relative power of the alpha band in the left parietal region was lower in the MDD group than in the HC group, which strongly contributed to the automatic classification of MDD. Previous studies suggest that dysregulation of the left subparietal cortex in depressed patients may not only be associated with deficits in audiovisual integration but also with impaired memory and emotional processing deficits in patients with MDD [42]. Therefore, the above results of this machine learning are reasonable. The results further confirmed the dysfunction of the left parietal lobe in patients with MDD [42]. Moreover, the finding was in agreement with the statistically calculated differences in EEG features. In the two independent sample t-tests, there were group differences in all the above three indicators. However, the relative power of alpha in the left parietal region was not significantly different after FDR correction. The reason for this may be related to the small number of subjects. Therefore, to some extent, there was a significant difference in the relative power of the alpha band in the left top region between the two groups. Additionally, the top three features belong to the alpha band power. The alpha band power achieves a high classification accuracy, which is consistent with a previous study [43]. Finally, our results showed that feature set 1 alone achieves the highest classification accuracy, which was higher than that of the combination of two feature sets. We have known that the set 2 achieved the lower accuracy in the machine learning. After the calculations of functional connectivity of any two regions above, we found that the values of wPLIs in depression group were less than 0.5. It indicated that the scalp of functional connectivity of any two regions were relatively lower. Therefore, when we regarded the functional connectivity as feature set 2, we achieved accuracy which varied from 0.50 to 0.618. The causes may be owed to the low resolution of the functional connectivity of any two scalp brain regions in each band. When we combined set1 and set2 together, we didn’t achieve the highest accuracy in the machine learning. We considered that combining with the connectivity may cause interference, which affects the classification and thus reduces the accuracy. However, our results achieved an accuracy of 88.2%, which was lower than the results of the studies of Abdolkarim Saeedi [44] and Chien-Te Wu [45]. Abdolkarim Saeedi et al. developed E-KNN rather than KNN to give information on feature importance index and improve the results (an accuracy of 98.44%). Chien Te Wu et al. utilized the combination of the optimal feature subset and CK-SVM achieving an accuracy of 91.07% on the training set. In comparison, we analyzed the spectral power and functional connectivity and combined with using five machine-learning algorithms for classification. We speculated that the feature selection and the method of classifier algorithms were contributed to our lower accuracy.

Due to the impact of medication on the EEG and emotional state of MDD patients, we recruit first-episode MDD patients who did not take medication to rule out the impact of medication as an interfering factor. We explored the abnormalities of EEG features in MDD patients to clarify the EEG mechanism of depression in resting state. Combining with the machine learning, we would provide a theoretical basis for early recognition of depression and the application of neuro electrophysiological markers in clinical practice.

### Limitations and future research directions

Several limitations should be considered in future studies. First, the sample size was small, which may affect the stability performance in machine learning. Moreover, there may exist correlations between spectrum power and the severity of MDD. However, the correlations may not be significant as a result of the limited sample size. Therefore, although our results provide a strong reference direction for future studies, they need to be further verified through large samples. Second, although we use many measures to control overfitting, including cutting data to increase the sample size, cross-validation and feature selection techniques, our performance estimates are not completely unbiased. To obtain unbiased estimates of our model prediction performance, future studies should be based on independent datasets that were not used for model construction for validation. Third, because our study belonged to cross section study, we wouldn’t investigate the impact of LP-Alpha feature in MDD diagnosis. Therefore, we would explore the value of LP-Alpha in the diagnosis of MDD using longitude research in the future study. At last, in the future work, we should learn more the methods of machine learning like the study of Li et al [46] to improve the accuracy and explore the biomarker of MDD patients better.

## Conclusions

In conclusion, we found that there were significant changes in EEG features, which indicated that there were potential abnormalities in EEG neural oscillations in first-episode and drug-naïve patients with MDD. This may reflect cortical excitation, inhibition, and overactivity imbalance in patients with drug-free MDD. After the machine learning algorithm, we hypothesized that the relative power of the alpha band in the left parietal region may be a potential neurophysiological marker of MDD. Moreover, it may be used as an indicator to assist in the clinical diagnosis of MDD.

### Electronic supplementary material

Below is the link to the electronic supplementary material.

Supplementary Material 1: Detailed process of machine learning

Supplementary Material 2: Comparison of the relative power

## Data Availability

The datasets used and/or analyzed during the current study are available from the corresponding author on reasonable request.
