# Supplementary material for: Analysis of EEG features and study of automatic classification in first-episode and drug-naïve patients with major depressive disorder
Source: BMC Psychiatry. 2023 Nov 13;23:832. doi: 10.1186/s12888-023-05349-9 (PMC10644563; doi:10.1186/s12888-023-05349-9)
Supplement: Supplementary file 1 — Supplementary Material 1: Detailed process of machine learning [file 12888_2023_5349_MOESM1_ESM.doc]

**Supplementary Material 1: Detailed process of machine learning.**

Firstly, we used 1000 sampling points as a sample. Since the data sampling rate was down sampled to 500Hz, a sample should take 2 seconds. After the data cutting, the total sample size of the two groups was 19512. Then, 90% of the data was used for training and 10% for testing. In this study, we used two-step method: 1) parameter adjustment stage: we divided the validation set and the random_ State=42. We can find the appropriate parameters in the first stage, and report the training set, validation set, and test set to prove the effectiveness of parameter tuning; 2) the reporting phase: there was no division of the validation set. We trained the model by using the parameters found in the first stage. In order to fully utilize the data, we repeated 100 times and finally take the mean and standard deviation of the results on the test set.

In the model training process, all model hyperparameters are adjusted in the validation set by grid search. The optimal hyperparameters are selected to train the model. To evaluate the classification performance during grid search, we use built-in function in the Python module (GridSearchCV) in scikit-learn, applying a ten-fold cross-validation. This function uses different sets of hyperparameters and cross-validation schemes to evaluate the classification combinations.
