# Supplementary material for: Analysis of EEG features and study of automatic classification in first-episode and drug-naïve patients with major depressive disorder
Source: BMC Psychiatry. 2023 Nov 13;23:832. doi: 10.1186/s12888-023-05349-9 (PMC10644563; doi:10.1186/s12888-023-05349-9)
Supplement: Supplementary file 2 — Supplementary Material 2: Comparison of the relative power [file 12888_2023_5349_MOESM2_ESM.doc]

**Supplementary Material 2：Comparison of the relative power.**

Table S1. Comparison of the relative power of each scalp-brain region in each frequency band between the two groups.

|  | MDD  Mean±SD | HCs  Mean±SD | *p* | *FDRp* |
| --- | --- | --- | --- | --- |
| LF-delta | 0.45±0.13 | 0.43±0.12 | 0.538 | 0.63 |
| LF-theta | 0.21±0.08 | 0.20±0.05 | 0.806 | 0.83 |
| LF-alpha | 0.27±0.14 | 0.31±0.13 | 0.235 | 0.36 |
| LF-beta | 0.05±0.03 | 0.04±0.02 | 0.061 | 0.17 |
| LF-gamma | 0.02±0.02 | 0.01±0.01 | 0.148 | 0.26 |
| RF-delta | 0.44±0.12 | 0.43±0.11 | 0.568 | 0.63 |
| RF-theta | 0.21±0.07 | 0.20±0.05 | 0.728 | 0.79 |
| RF-alpha | 0.27±0.13 | 0.31±0.13 | 0.279 | 0.37 |
| RF-beta | 0.05±0.03 | 0.04±0.02 | 0.072 | 0.19 |
| RF-gamma | 0.02±0.01 | 0.01±0.01 | 0.374 | 0.47 |
| LP-delta | 0.40±0.13 | 0.34±0.12 | 0.072 | 0.19 |
| LP-theta | 0.18±0.06 | 0.17±0.05 | 0.359 | 0.46 |
| LP-alpha | 0.34±0.16 | 0.43±0.14 | 0.015 | 0.09 |
| LP-beta | 0.06±0.03 | 0.04±0.02 | 0.022 | 0.09 |
| LP-gamma | 0.02±0.02 | 0.01±0.01 | 0.048 | 0.16 |
| RP-delta | 0.41±0.14 | 0.33±0.11 | 0.019 | 0.1 |
| RP-theta | 0.18±0.06 | 0.16±0.05 | 0.246 | 0.36 |
| RP-alpha | 0.33±0.16 | 0.44±0.15 | 0.004 | ***0.03*** |
| RP-beta | 0.06±0.03 | 0.05±0.02 | 0.059 | 0.18 |
| RP-gamma | 0.02±0.02 | 0.01±0.01 | 0.267 | 0.38 |
| LT-delta | 0.46±0.13 | 0.42±0.11 | 0.145 | 0.26 |
| LT-theta | 0.17±0.06 | 0.18±0.05 | 0.552 | 0.63 |
| LT-alpha | 0.24±0.12 | 0.31±0.13 | 0.038 | 0.14 |
| LT-beta | 0.08±0.06 | 0.06±0.03 | 0.174 | 0.29 |
| LT-gamma | 0.05±0.05 | 0.04±0.03 | 0.274 | 0.38 |
| RT-delta | 0.47±0.14 | 0.42±0.10 | 0.098 | 0.22 |
| RT-theta | 0.17±0.05 | 0.18±0.05 | 0.519 | 0.63 |
| RT-alpha | 0.25±0.12 | 0.30±0.11 | 0.111 | 0.22 |
| RT-beta | 0.07±0.05 | 0.07±0.05 | 0.739 | 0.78 |
| RT-gamma | 0.04±0.05 | 0.04±0.04 | 0.968 | 0.97 |
| LO-delta | 0.37±0.18 | 0.25±0.09 | 0.002 | ***0.02*** |
| LO-theta | 0.15±0.06 | 0.12±0.04 | 0.02 | 0.09 |
| LO-alpha | 0.36±0.18 | 0.54±0.13 | 0.001 | ***0.02*** |
| LO-beta | 0.08±0.06 | 0.05±0.03 | 0.079 | 0.19 |
| LO-gamma | 0.04±0.05 | 0.02±0.02 | 0.117 | 0.22 |
| RO-delta | 0.41±0.17 | 0.23±0.10 | 0.001 | ***0.02*** |
| RO-theta | 0.15±0.05 | 0.11±0.03 | 0.001 | ***0.02*** |
| RO-alpha | 0.33±0.16 | 0.58±0.16 | 0.001 | ***0.02*** |
| RO-beta | 0.07±0.05 | 0.05±0.03 | 0.103 | 0.22 |
| RO-gamma | 0.03±0.04 | 0.02±0.03 | 0.187 | 0.3 |

MDD: major depression disorder; HCs: Healthy controls; SD: Standard deviation; LF/RF/LP/RP/LT/RT/LO/RO-delta/theta/alpha/beta/gamma: Left Frontal/ Right Frontal/ Left Parietal/ Right Parietal/ Left Temporal/ Right Temporal/ Left Occipital / Right Occipital-delta/ theta/ alpha/ beta/ gamma
